# Chronic Occupational Mold Exposure Drives Expansion of *Aspergillus*-Reactive Type 1 and Type 2 T-Helper Cell Responses

**DOI:** 10.3390/jof7090698

**Published:** 2021-08-27

**Authors:** Chris D. Lauruschkat, Sonja Etter, Elisabeth Schnack, Frank Ebel, Sascha Schäuble, Lukas Page, Dana Rümens, Mariola Dragan, Nicolas Schlegel, Gianni Panagiotou, Olaf Kniemeyer, Axel A. Brakhage, Hermann Einsele, Sebastian Wurster, Juergen Loeffler

**Affiliations:** 1 Department of Internal Medicine II, University Hospital of Wuerzburg, 97080 Wuerzburg, Germany; Lauruschka_c@ukw.de (C.D.L.); sonja.etter@stud-mail.uni-wuerzburg.de (S.E.); E_Page_L@ukw.de (L.P.); dana_alicia.ruemens@stud-mail.uni-wuerzburg.de (D.R.); Einsele_H@ukw.de (H.E.); 2 Institute for Infectious Diseases and Zoonoses, Ludwig-Maximilians-University of Munich, 80539 Munich, Germany; Elisabeth.Schnack@micro.vetmed.uni-muenchen.de (E.S.); frank.ebel@lmu.de (F.E.); 3 Systems Biology and Bioinformatics, Leibniz Institute for Natural Product Research and Infection Biology—Hans-Knoell-Institute (HKI), 07745 Jena, Germany; sascha.schaeuble@hki-jena.de (S.S.); gianni.panagiotou@hki-jena.de (G.P.); 4 Department of Surgery I, University Hospital of Wuerzburg, 97080 Wuerzburg, Germany; Dragan_M@ukw.de (M.D.); schlegel_n@ukw.de (N.S.); 5 Department of Microbiology, Li Ka Shing Faculty of Medicine, The University of Hong Kong, Hong Kong, China; 6 Department of Molecular and Applied Microbiology, Leibniz Institute for Natural Product Research and Infection Biology—Hans-Knoell-Institute (HKI), 07745 Jena, Germany; Olaf.Kniemeyer@hki-jena.de (O.K.); axel.brakhage@leibniz-hki.de (A.A.B.); 7 Department of Infectious Diseases, Infection Control and Employee Health, The University of Texas MD Anderson Cancer Center, Houston, TX 77030, USA; STWurster@mdanderson.org

**Keywords:** mold exposure, immunoassay, biomarker, *Aspergillus*, cytokines, inflammation, adaptive immunity, hypersensitivity

## Abstract

Occupational mold exposure can lead to *Aspergillus*-associated allergic diseases including asthma and hypersensitivity pneumonitis. Elevated IL-17 levels or disbalanced T-helper (Th) cell expansion were previously linked to *Aspergillus*-associated allergic diseases, whereas alterations to the Th cell repertoire in healthy occupationally exposed subjects are scarcely studied. Therefore, we employed functional immunoassays to compare Th cell responses to *A. fumigatus* antigens in organic farmers, a cohort frequently exposed to environmental molds, and non-occupationally exposed controls. Organic farmers harbored significantly higher *A. fumigatus*-specific Th-cell frequencies than controls, with comparable expansion of Th1- and Th2-cell frequencies but only slightly elevated Th17-cell frequencies. Accordingly, *Aspergillus* antigen-induced Th1 and Th2 cytokine levels were strongly elevated, whereas induction of IL-17A was minimal. Additionally, increased levels of some innate immune cell-derived cytokines were found in samples from organic farmers. Antigen-induced cytokine release combined with *Aspergillus*-specific Th-cell frequencies resulted in high classification accuracy between organic farmers and controls. Aspf22, CatB, and CipC elicited the strongest differences in Th1 and Th2 responses between the two cohorts, suggesting these antigens as potential candidates for future bio-effect monitoring approaches. Overall, we found that occupationally exposed agricultural workers display a largely balanced co-expansion of Th1 and Th2 immunity with only minor changes in Th17 responses.

## 1. Introduction

Humans inhale hundreds of airborne spores of environmental molds daily such as *Aspergillus* species [1]. While healthy individuals can efficiently clear fungal spores from their lungs, chronic mold exposure is a major risk factor for the development of hypersensitivity. Dysregulation of the immune system due to long-term exposure to high concentrations of *Aspergillus* spores can lead to severe asthma with fungal sensitization, allergic sinusitis, allergic bronchopulmonary aspergillosis (ABPA), and hypersensitivity pneumonitis [2,3,4]. Of note, a significant proportion of *Aspergillus*-associated allergic diseases are seen in occupationally exposed patients such as agricultural workers [2,5,6,7,8]. However, in contrast to serological responses, little is known about the effects of intensive and chronic occupational mold exposure on the human *Aspergillus*-reactive T-(helper) cell repertoire.

Recent advances in functional immunoassays have improved our understanding of mold-antigen reactive T-cell subsets in diverse patient populations with *Aspergillus*-associated diseases and individuals with varying degrees of mold exposure. Specifically, we and others have previously shown that environmental exposure to molds and mycotoxins can result in assayable alterations to the human mold-reactive T-cell repertoire and proinflammatory cytokine release [9,10,11]. However, these studies have been performed in predominantly residentially exposed subjects who had a relatively low risk of *Aspergillus*-associated diseases, whereas data regarding the specific impact of occupational mold exposure on *Aspergillus fumigatus*-antigen reactive T-cell expansion, polarization, and cytokine responses are scarce.

Therefore, we employed flow cytometry, enzyme-linked immunospot assays (ELISPOT), and a recently developed whole-blood-based cytokine release assay [12] with dual T-cellular co-stimulation in order to compare T-helper (Th) cell responses to mold antigens in a defined occupationally mold-exposed cohort and non-occupationally exposed controls. We selected agricultural workers as our occupationally exposed cohort due to their known risk for extensive *Aspergillus* exposure, mold sensitization, and development of asthma and hypersensitivity pneumonitis (“farmer’s lung”) [5,6,13]. In particular, organic farming approaches that, among other restrictions, omit the use of azoles and other chemical fungicides, can result in higher abundance of *A. fumigatus* compared to conventional production [14].

Although methodologies for recombinant expression of *Aspergillus* proteins have been considerably improved and some antigens are commercially available, systematic studies of exposure-dependent T-cell responses to individual *A. fumigatus* antigens have not been performed thus far [15]. Hence, the most potent *A. fumigatus* antigens for the development of bio-effect monitoring strategies by means of functional T-cell assays remain to be defined. To that end, we analyzed *A. fumigatus* antigen-reactive type-1 (Th1), type-2 (Th2), and type-17 (Th17) Th cell responses to a mycelial lysate and 12 proteinaceous *A. fumigatus* antigens in samples from organic farmers and controls and describe discriminative Th cell frequencies as well as *A. fumigatus* driven Th1, Th2, and Th17 associated cytokine releases.

## 2. Materials and Methods

### 2.1. Subjects

The occupationally exposed cohort included 9 male and 1 female organic farmer(s) aged 18–70 (median: 30) who have worked in the agricultural industry for 1 to 30 years (median: 9) and were sampled within three months after the harvesting period. Organic farming approaches were verified by the following certifications documenting consistency of agricultural practices with the European Council regulation on organic production and labelling of organic products (834/2007): Demeter (DE-ÖKO-022), Naturland, A-B-CERT (DE-ÖKO-06), and/or Kontrollverein ökologischer Anbau e.V. (DE-ÖKO-022). The control cohort consisted of 4 male and 6 female healthy blood donors aged 22–65 (median: 25). Exclusion criteria for both cohorts were pregnancy, acute infections or recent antimicrobial therapy (within the last 12 weeks), and allergic diseases requiring immunomodulatory treatment (within the last 12 weeks).

### 2.2. Blood Collection and PBMC Isolation

Venous blood was drawn into Monovette^®^ lithium heparin blood collection tubes (Sarstedt, Newton, USA). Peripheral blood mononuclear cells (PBMC) were isolated using Leucosep™ tubes (Greiner Bio-One, Frickenhausen, Germany) and Biocoll^®^ separation solution (1.077 g/mL, Merck, Darmstadt, Germany). After separation, PBMCs were washed with 45 mL of CTL Wash™ (CTL Europe, Bonn, Germany) and suspended in 1 mL of CTL Wash™. Cells were counted with a Vi-Cell XR counter (Beckman Coulter, Brea, USA) and divided for ELISPOT and flow cytometry. After centrifugation, PBMCs for flow cytometry were resuspended at a concentration of 1 × 10^7^ cells per mL in RPMI 1640 Glutamax™ (Gibco, Thermo Fisher, Waltham, USA) supplemented with 5% heat-inactivated, sterile-filtered autologous serum. PBMCs for ELISPOT were diluted in CTL Test™ medium (CTL Europe, Bonn, Germany) at a concentration of 2 × 10^6^ cells per mL.

### 2.3. Aspergillus Fumigatus Mycelial Lysate and Recombinant Antigen Generation

An *A. fumigatus* mycelial lysate (AfuLy) and proteinaceous antigens were generated as described before [12]. Primer sequences for the amplification of fungal cDNA are provided in Table S1.

### 2.4. ELISPOT

IFN-γ, IL-5, and IL-17 ELISPOT assays (Lophius Biosciences, Regensburg, Germany) were performed according to the manufacturer’s recommendations, with minor modifications as described before [16]. Briefly, 2 × 10^5^ PBMCs were added to each well of the ELISPOT plate and stimulated with AfuLy (50 μg/mL) or proteinaceous antigens (30 μg/mL). Plates for IFN-γ or IL-5 ELISPOTs were supplemented with 1 μg/mL α-CD28 and 1 μg/mL α-CD49d co-stimulatory antibodies (both Miltenyi Biotec, Bergisch Gladbach, Germany), whereas no co-stimulatory antibodies were used for IL-17 ELISPOTs, following previously optimized protocols [17]. Phytohemagglutinin (PHA, 10 μg/mL, Sigma-Aldrich, St. Louis, MI, USA) served as a positive control. Unstimulated control wells contained PBMCs and co-stimulatory factors (if applicable), but no antigens. IFN-γ ELISPOTs were incubated for 24–26 h at 37 °C and 5 % CO_2_, whereas longer incubation (44–48 h) was used for IL-5 and IL-17 ELISPOTs. Staining was performed according to the manufacturer’s instructions and numbers of spot-forming cells (SFCs) per million PBMCs were determined with a Bioreader 5000a (BioSYS, Karben, Germany), using previously published readout settings [17].

### 2.5. Flow Cytometry

Flow cytometry was performed as described before [18]. α-CD8-FITC, α-CD196 (CCR6)-APC, α-CD197 (CCR7)-PE, α-CD4-VioBlue, α-CD183 (CXCR3)-PE-Vio615, α-CD45RA-VioGreen, α-CD154-PE-Vio770, α-IFN-γ-APC-Vio770, and 7-aminoactinomycin D staining solution (all Miltenyi Biotec, Bergisch Gladbach, Germany) were used for cell staining in combination with the Inside Stain Kit (Miltenyi Biotec, Bergisch Gladbach, Germany). All dyes were stained extracellularly, except for the intracellular markers CD154 and IFN-γ. Samples were measured using a CytoFLEX cytometer and CytExpert software (Beckman-Coulter, Brea, USA). Data were analyzed with FlowJo 10.6.1. The gating strategy and a representative dataset are shown in Figure S1.

### 2.6. Whole-Blood Stimulation for Multiplex Cytokine Assays

Anticoagulant-free stimulation tubes were prepared as detailed in Table S2, following a previously published protocol [12], and cryopreserved at −20 °C for up to four weeks. Prior to whole-blood (WB) stimulation, the ready-to-use stimulation tubes were brought to room temperature. Within 90 min of blood collection, 500 µL of WB was injected into the tubes with a graduated 1-mL insulin syringe. Stimulation tubes were inverted 10 times and incubated for 24 h at 37 °C. Plasma was collected by centrifugation at 2000× *g* for 20 min and cryopreserved at −20 °C. Cytokine concentrations were determined using a 21-plex Milliplex^®^ MAP human high-sensitivity T-cell magnetic bead panel kit (Merck, Darmstadt, Germany) and a Luminex200 reader (Luminex, Austin, TX, USA) in combination with the XPONENT3 (Luminex, Austin, TX, USA) and Milliplex Analyte software (Merck, Darmstadt, Germany). Cytokine concentrations were interpolated from 7-point standard curves.

### 2.7. Statistical Analyses

All immunoassay results presented in this manuscript were adjusted for unspecific background reactivity by subtraction of activation marker expression or cytokine release detectable in unstimulated samples. The Mann–Whitney-U-test with or without subsequent Benjamini–Hochberg correction for a false-positive discovery rate (FDR) of 0.2 was applied for significance testing (as specified in the figure legends). The correlation of results across different assays was evaluated by Spearman’s rank correlation coefficients. The classification of organic farmers and controls based on immunoassay results was accomplished by optimizing a random forest machine learning model using the caret (v6.0) package in the R statistical programming environment (v4.1). Combinations of up to three cytokine analytes were tested with and without consideration of *A. fumigatus*-specific T-cell frequencies. Caret’s train and trainControl functions were applied for random forest optimization using 5-fold cross-validation that was repeated 100 times based on center-scaled input data. Overall accuracy across all repeated cross-validation runs was reported as model performance. Data were analyzed and visualized using GraphPad Prism version 8 and Microsoft Excel.

## 3. Results

**Figure 1:**
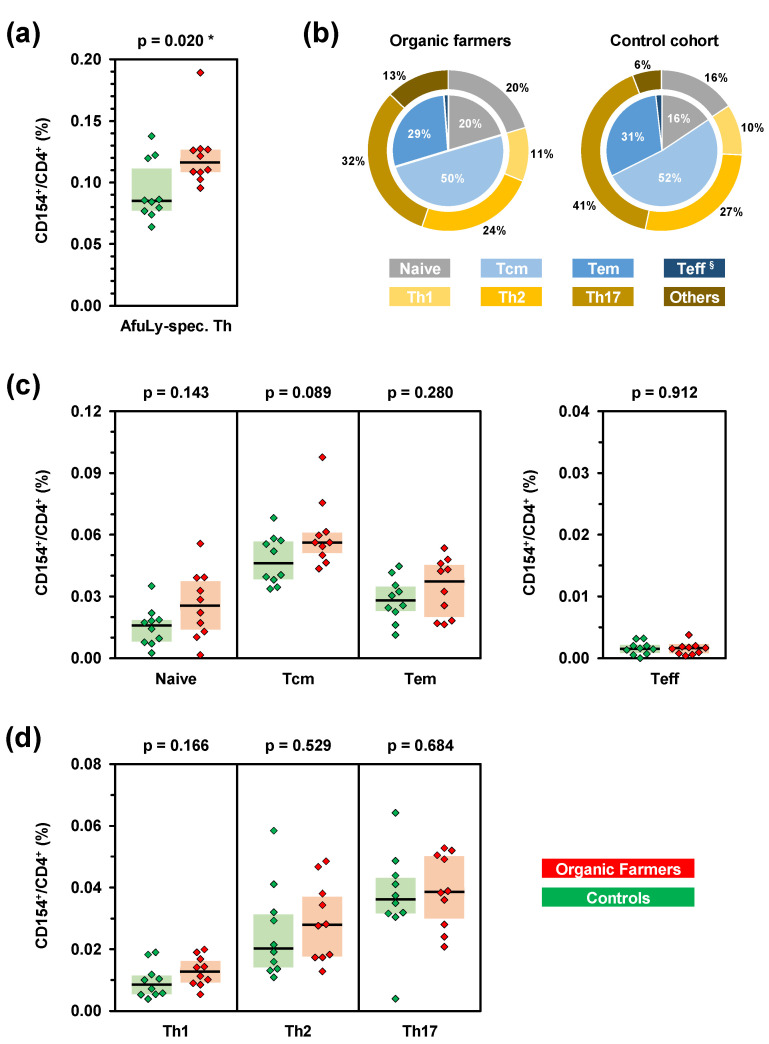
Subjects with occupational mold exposure harbor increased frequencies of *A. fumigatus*-specific T-helper (Th) cells. PBMCs from 10 organic farmers (red) and 10 non-occupationally exposed control subjects (green) were stimulated with *A. fumigatus* mycelial lysate (AfuLy). Frequencies and phenotypes of CD154^+^ Th cells (CD4^+^ cells) were determined by flow cytometry. (**a**) Total background-adjusted AfuLy-specific Th cell frequencies. (**b**) Mean percentages of effector/memory phenotypes (inner pie) and Th cell lineages (outer ring) among CD4^+^CD154^+^ AfuLy-specific Th cells in organic farmers and controls. Tcm = central memory Th cells, Tem = effector memory Th cells, Teff = effector Th cells (^§^ 1% in organic farmers, 2% in controls). (**c**,**d**) Frequencies of AfuLy-reactive CD154^+^ effector/memory Th cell populations (**c**) and Th1/Th2/Th17 cells (**d**) among CD4^+^ Th cells in organic farmers and controls. (**a**,**c**,**d**) Background-adjusted individual values, medians (black bars), and inter-quartile ranges (colored boxes) are shown. Two-sided Mann–Whitney-U-test. Asterisks indicate significant differences (*p* < 0.05).

At first, we performed flow cytometry to determine the total *A. fumigatus* reactive Th cell repertoire of organic farmers and subjects without occupational mold exposure. The organic farmer cohort had significantly greater median CD154^+^ AfuLy-specific Th cell frequencies (0.116%) than the control cohort (0.085%, *p* = 0.020, Figure 1a). Consistent with our previously published results [9,10], all three control subjects with more than 0.1% *A. fumigatus*-specific Th cells had significant residential mold exposure (“high exposure”), as determined using a previously validated questionnaire to capture surrogates of mold encounter such as close proximity of the place of residence to farmlands [10] (Figure S2). In contrast, five out of seven control subjects with *A. fumigatus*-specific T-cell frequencies of <0.1% had a “low exposure” profile as defined in reference [10].

When assessing effector and memory phenotypes of *A. fumigatus*-specific Th cells by CCR7 and CD45RA expression, no significant differences in relative distributions of phenotypes were found between organic farmers and controls (Figure 1b). Instead, all subsets except effector Th cells were moderately yet non-significantly expanded in the organic farmer cohort (Figure 1c). Similarly, no significant elevations of individual Th cell lineages were found in the organic farmer cohort (Figure 1b,d). However, both median Th1 (+47%) and Th2 (+38%) cell frequencies were modestly expanded in organic farmers, whereas median AfuLy-reactive Th17 cell frequencies differed by only 7% between the two cohorts (Figure 1d).

**Figure 2:**
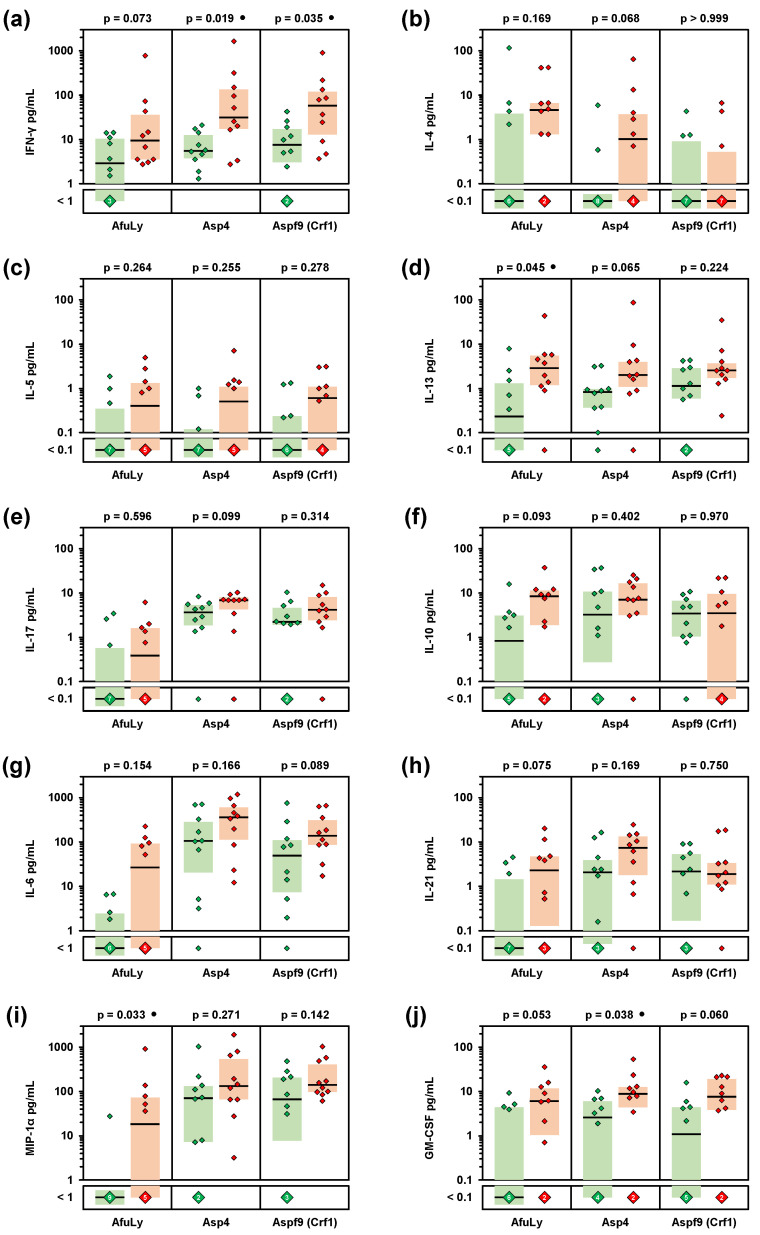
Organic farmers show enhanced Th1, Th2, and innate immune cell-derived cytokine responses to *A. fumigatus* antigens. Whole-blood samples from 10 organic farmers (red) and 10 non-occupationally exposed controls (green) were stimulated with *A. fumigatus* mycelial lysate (AfuLy), Aspf4, or Aspf9/Crf1. Cytokine concentrations in plasma supernatants were quantified using a Luminex assay. Individual and median (black bars) background-adjusted cytokine concentrations of INF-γ (**a**), IL-4 (**b**), IL-5 (**c**), IL-13 (**d**), IL-17 (**e**), IL-10 (**f**), IL-6 (**g**), IL-21 (**h**), MIP-1α (**i**), and GM-CSF (**j**) are shown. Colored boxes represent inter-quartile ranges. Two-sided Mann–Whitney-U-test with Benjamini–Hochberg correction for an FDR of 0.2. No comparison reached FDR-corrected significance between organic farmers and controls (*p* < 0.05, FDR < 0.2). Black circles indicate a trend toward significance (*p* < 0.05, FDR > 0.2).

Next, we employed a WB-based cytokine assays to quantify the release of 21 cytokines and chemokines in response to AfuLy and the well-characterized *A. fumigatus* allergens Asp4 and Aspf9/Crf1 [19,20,21,22,23,24]. Although not reaching statistical significance after correction for multiple testing, all three stimuli elicited 3.3–7.6-fold greater median IFN-γ release in samples from organic farmers compared to controls (Figure 2a and Figure S3a–c). Similarly, elevations of the Th2 cytokines IL-4, IL-5,2 and, in particular, IL-13 were found in the organic farmer cohort (Figure 2b,d and Figure S3a–c). In contrast, differences in IL-17 response between the two cohorts were relatively minor (median-median-ratio ≤1.9, Figure 2e and Figure S3a–c). Compared to controls, organic farmers also showed 10-fold greater median release of the regulatory T-cell (Treg) signature cytokine IL-10 in AfuLy-stimulated WB (Figure 2f and Figure S3a). Furthermore, strongly enhanced release (≥10-fold median-to-median ratio) of several cytokines predominantly derived from antigen-presenting cells (APCs), especially GM-CSF, MIP-1α, IL-6, and IL-21, was found in AfuLy-stimulated WB from organic farmers versus controls (Figure 2g–j and Figure S3a). Although less pronounced, similar trends were found for Aspf4 and Crf1 stimulation (Figure S3b,c). Collectively, these data indicate that occupational mold exposure triggers enhanced Th1 and Th2 cytokine release as well as increased APC activation in response to *A. fumigatus* antigens.

**Figure 3:**
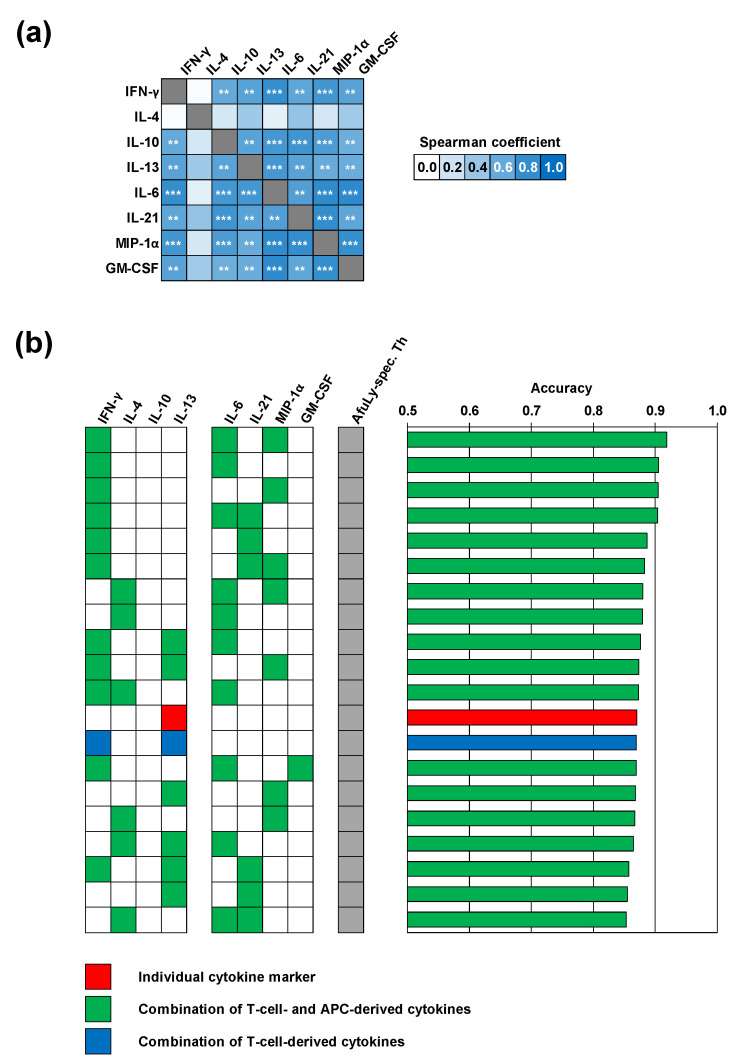
Machine learning corroborates a selection of highly correlated T-cellular and APC-derived cytokine responses with robust discriminatory power between occupationally exposed subjects and controls. (**a**) Heatmap of Spearman’s rank correlation coefficients of individual cytokine concentrations elicited by *A. fumigatus* mycelial lysate (AfuLy, Luminex assay results). FDR-corrected significance of Spearman coefficients is indicated by asterisks. ** *p* < 0.01, *** *p* < 0.001. (**b**) Random forest analysis to determine the classification accuracy of combinations of up to three cytokine responses to AfuLy along with flow cytometrically determined CD154^+^ AfuLy-reactive T-helper cell frequencies. The top 20 combinations with the highest classification accuracy between organic farmers and controls are shown. Only cytokine markers passing the pre-filtering step (Figure S4) based on their relative induction in the two cohorts (median-to-median ratio > 2.0) and *p*-value (*p* < 0.2) were considered for both panels.

Considering both median-to-median ratios between the two cohorts and statistical significance, we identified a selection of eight cytokines that displayed the strongest differences in *A. fumigatus* antigen-induced release between the two cohorts (Figure S4). Cytokines with markedly stronger induction by AfuLy in organic farmers than in controls included the Th1 cytokine INF-γ, the Th2 cytokines IL-4 and IL-13, the Treg cytokine IL-10, and the innate immune cell-derived cytokines IL-6, IL-21, MIP-1α, and GM-CSF (Figure S4). Seven out of the eight cytokines were also identified as differently induced by Aspf4 and/or Aspf9/Crf1 (Figure S4). Based on this pre-selection, we found strong and significant positive correlation of individual AfuLy-reactive T-cellular cytokine responses, but also strong positive correlation between T-cellular and APC cytokine release (Figure 3a; all assayed cytokines are shown in Figure S5). These results indicate co-induction of antigen-reactive Th1 and Th2 responses in the organic farmer cohort and point to a role of highly correlated innate immune cell-derived cytokines in shaping *A. fumigatus*-reactive immune responses in subjects with extensive occupational mold exposure.

We then employed a machine learning algorithm using random forest classification to further narrow down the cytokines with the best discriminatory power between organic farmers and controls. Four combinations of AfuLy-elicited cytokines with modest discriminatory power (>0.75) were found, all of them containing the Th1 cytokine INF-γ along with cytokines derived from innate immune cells, with or without the Th2 cytokine IL-4 (Figure S6). In line with our previously published results [9], classification accuracy between samples from organic farmers and controls strongly improved when combining AfuLy-reactive Th cell frequencies and up to three cytokine markers (Figure 3b). Despite the heterogeneity of the control cohort, the combination of AfuLy-reactive Th cell frequencies with IFN-γ, IL-6, and MIP-1α release resulted in a classification accuracy of 92%, suggesting potential usefulness of these cytokine markers for bio-effect monitoring strategies in occupationally exposed subjects. Of note, all the top 11 most accurate combinations (classification accuracy 87–92%) contained a Th1 (IFN-γ) and/or Th2 cytokine (IL-4) in combination with at least one of the APC-derived cytokines IL-6, IL-21, and MIP-1α (Figure 3b).

**Figure 4:**
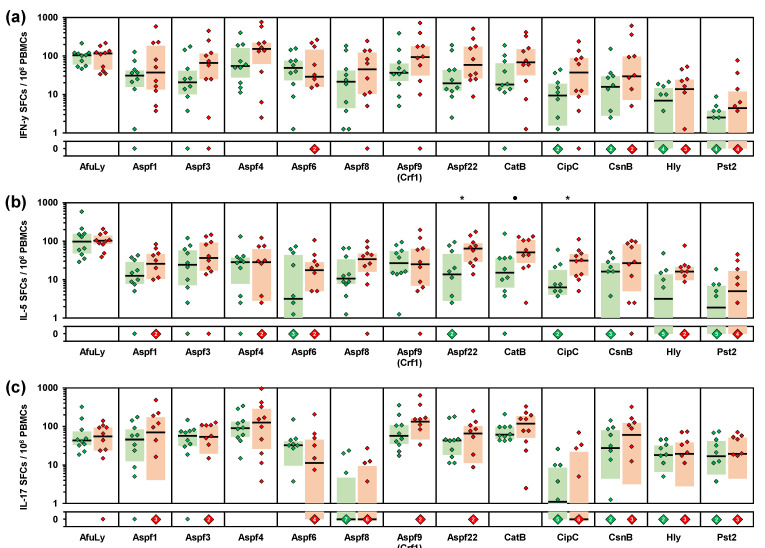
PBMCs from organic farmers display enhanced Th1 and Th2 responses to selected *A. fumigatus* antigens compared with non-occupationally exposed controls. PBMCs from 10 organic farmers (red) and 10 non-occupationally exposed control subjects (green) were stimulated with *A. fumigatus* mycelial lysate (AfuLy) or proteins. Numbers of IFN-γ (**a**), IL-5 (**b**), and IL-17 (**c**) producing cells (spot forming cells, SFCs) per million PBMCs were determined by ELISPOT. Individual background-adjusted SFC counts, medians (black bars), and inter-quartile ranges (colored boxes) are shown. Two-sided Mann–Whitney-U-test with Benjamini–Hochberg correction for an FDR of 0.2. Asterisks indicate significant differences between organic farmers and controls (*p* < 0.05, FDR < 0.2). Black circles indicate a trend toward significance (*p* < 0.05, FDR > 0.2).

Next, we sought to characterize exposure-driven differences in immune responses to individual *A. fumigatus* antigens. To that end, we used ELISPOT assays to compare the frequencies of cells secreting Th1 (IFN-γ), Th2 (IL-5), and Th17 (IL-17) signature cytokines after stimulation with AfuLy and 12 proteinaceous *A. fumigatus* antigens (Figure 4). Although not reaching statistical significance due to the limited number of subjects, eight antigens (Aspf3, Aspf4, Aspf8, Aspf9, Aspf22, CatB, CipC, and Hly) elicited at least 2-fold higher IFN-γ SFC counts in PBMCs from organic farmers compared with controls (Figure 4a). Similarly, eight antigens (Aspf1, Aspf6, Aspf8, Aspf22, CatB, CipC, Hly, and Pst2) caused >2-fold greater IL-5 responses of PBMCs from organic farmers versus controls (Figure 4b).

**Figure 5:**
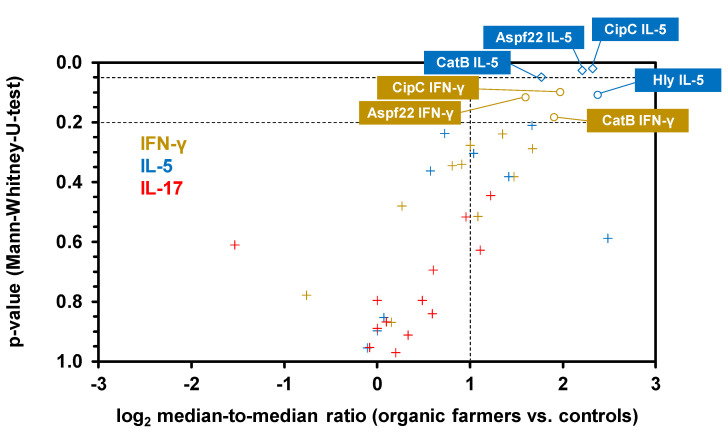
ELISPOT reveals a cluster of proteinaceous *A. fumigatus* antigens that elicit enhanced Th1 and Th2 signature cytokine response in subjects with occupational mold exposure. Volcano plot combining log_2_-transformed ratios of median SFCs counts for each antigen/cytokine pair in organic farmers and controls with the corresponding *p*-values (Mann–Whitney-U-test). Raw data are derived from Figure 4. Antigen/cytokine pairs are classified as insignificant (“+” symbols, median-to-median ratio <2 and/or *p* > 0.2), potentially significant (circles, median-to-median ratio > 2 and 0.05 < *p* < 0.2), or significant (diamonds, median-to-median ratio >2 and *p* < 0.05). Antigen/cytokine pairs with medians of 0 SFCs in both cohorts are not displayed.

A volcano plot analysis combining the relative median SFC counts in the two cohorts with non-parametric significance testing revealed that Aspf22, CipC, and CatB elicited the most strongly distinct Th1 and Th2 responses between the two cohorts (Figure 5). Consistent with the results of our multiplex cytokine assays, ELISPOT showed minimal differences in IL-17 responses between the two cohorts, with only two antigens (Aspf1/Crf9 and CnsB) eliciting ≥2-fold greater median SFC counts in samples from organic farmers than in control samples (Figure 4c and Figure 5). Altogether, these data further corroborate the co-induction of Th1 and Th2 but not Th17 signature cytokine responses to selected *A. fumigatus* antigens in healthy, occupationally mold-exposed individuals.

**Figure 6:**
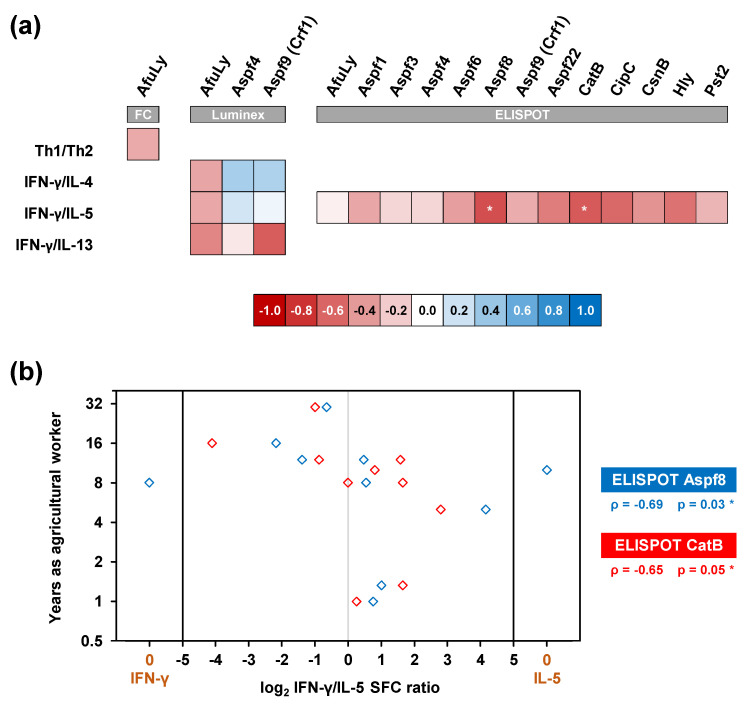
Chronic occupational mold exposure moderately correlates with a Th2-polarized immune phenotype. (**a**) Heatmap of Spearman’s rank correlation coefficients between the duration of service in organic farming and individual Th1/Th2 ratios (flow cytometry, FC) or ratios of Th1 (IFN-γ) and Th2 (IL-4, IL5, and IL-13) signature cytokine release detectable by WB-based Luminex analysis and ELISPOT after stimulation with *A. fumigatus* antigens. * *p* < 0.05. (**b**) Individual log_2_-transformed ratios of IFN-γ and IL-5 spot forming cell counts (SFC) upon stimulation with Aspf8 and CatB (x-value) compared with the subjects’ duration of service in organic farming (y-value). Spearman’s rank correlation coefficients (ρ) and their two-sided *p*-values are given.

Lastly, we tested whether our findings represent global co-induction of Th1 and Th2 responses in organic farmers or whether agricultural workers with longer durations of service/employment, and thus, more chronic exposure, display a distinct phenotype. Interestingly, across all three assay platforms, we consistently observed weak to moderate negative correlation between the number of years worked in organic farming and the Th1/Th2 balance (Figure 6a), suggesting stronger Th2 expansion in longer-serving farmers. Given the small cohort size, this observation only reached statistical significance for cytokine responses to Aspf8 and CatB in ELISPOT analyses (Figure 6b). Nonetheless, these data indicate some nuances to Th1/Th2 co-induction in our organic farmer cohort, with a trend toward stronger Th2 polarization in subjects with more chronic occupational mold exposure.

## 4. Discussion

Occupational mold exposure is associated with the development of asthma and hypersensitivity pneumonitis and can worsen symptoms of a wide range of allergic diseases [2,5,6,7,8]. Given its small, respirable conidial size and ubiquitous presence in the environment, *A. fumigatus* is responsible for a significant portion of mold-associated allergic diseases [2,3,4]. In previous studies, we have shown that *A. fumigatus* exposure contributes to elevated frequencies of *A. fumigatus* specific T cells detectable by flow cytometry or cytokine assays [9,10]. However, in these studies, we examined a heterogeneous group of donors with residential and occupational exposures, who mostly had no risk for *Aspergillus*-associated hypersensitivity diseases. In order to better understand the effects of chronic occupational mold exposure, we herein studied *A. fumigatus*-reactive Th cell responses of organic farmers, a cohort at risk for *Aspergillus*-associated hypersensitivity diseases [5,6,13], and compared the results to those of non-occupationally exposed subjects.

Expectedly, organic farmers had significantly higher CD154^+^ AfuLy-specific Th-cell frequencies than non-occupationally exposed individuals. However, specific Th-cell frequencies of occupationally exposed organic farmers overlapped with those of subjects reporting residential exposure. Thus, chronic occupational mold exposure does not necessarily lead to strongly elevated specific Th-cell frequencies, hinting at regulatory mechanisms that prevent an excessive immune response to high spore exposure and, eventually, hypersensitivity diseases. In fact, Treg cells were shown to play an important role in the modulation of adaptive immunity to *A. fumigatus* [25]. Although not the focus of this study, we found that AfuLy-stimulated WB samples from organic farmers produced 10-fold greater levels of the Treg signature cytokine IL-10 than those from controls, while IL-10 release was strongly correlated with elevated Th1 and Th2 responses.

Consistent with animal studies showing that extensive or serial exposure of healthy mice or piglets to *A. fumigatus* conidia led to a co-expansion of various Th cell subsets [26,27], our cohort of organic farmers without known mold-associated hyperinflammatory diseases displayed a comparable increase in AfuLy-reactive Th1 and Th2 cells. Accordingly, ELISPOT and WB-based Luminex assays revealed marked induction of *A. fumigatus* antigen-reactive Th1- and Th2-cell-derived cytokines in organic farmers, whereas differences in IL-17 levels were much smaller between the two cohorts. In contrast to these findings in our healthy mold-exposed cohort of agricultural workers, we and others previously reported that patients with *Aspergillus-*associated hypersensitivity diseases, such as ABPA, display strongly elevated Th17 cytokine responses to *A. fumigatus* antigens [12,28]. Although important in the initial stage of fungal clearance [29,30,31,32], enhanced Th17-cell activation has been associated with *Aspergillus* airway colonization and fungal persistence and is considered a driving factor in the establishment of hypersensitivity pneumonitis, a potential complication of chronic occupational mold exposure [28,33,34,35,36,37].

Our results further indicate that increased Th2 cytokine levels in response to *Aspergillus* antigens *per se* do not constitute a pathological value if they are accompanied by protective responses (that is, Th1-cell cytokines), whereas Th2-skewed immune responses without adequate Th1 expansion are associated with fungal persistence and allergic diseases, e.g., asthma and ABPA [38,39,40,41]. However, we observed a trend toward greater Th2 induction in subjects with chronic occupational mold exposure, which appears to precede serologically detectable mold sensitization (data not shown).

Compared to PBMC-based assays, WB provides a more physiological stimulation environment, allowing us to capture feedback loops between T cells and innate immune cells that can enhance cytokine responses [12]. This might explain why Crf1 and Aspf4, which we selected for our WB-based Luminex assay due to their known allergenic properties [19,20,21,22,23,24], elicited stronger differences in cytokine responses between organic farmers and controls in the Luminex panel than in the ELISPOT assay. Unlike our previous PBMC-based study that found predominantly increased T-cellular cytokine responses in mold-exposed subjects [9], WB stimulation with *A. fumigatus* antigens also induced elevated concentrations of several innate immune cell-derived cytokines in samples from organic farmers. In fact, the 11 most accurate combinations identified by machine learning featured at least one of the innate immune cell markers IL-6, IL-21, and/or MIP-1α.

Interestingly, IL-6, IL-21, and MIP-1α have well-characterized roles in shaping Th-cell polarization. For instance, IL-6 inhibits Treg and Th1 activity, supports the differentiation of naive T cells into Th2 and Th17 cells, and can induce IL-4 and IL-13 production by Th2 cells [42,43,44,45,46]. Conversely, MIP-1α induces Th1 polarization and was shown to prevent the switch from protective Th1 to non-protective Th2 responses to fungal pathogens [47]. Furthermore, the MIP-1α/MIP-1β-CCR5 axis is the predominant mechanism of Th-cell and cytotoxic T-cell recruitment by Treg cells in order to suppress effector T-cell activity [48], thereby potentially contributing to protective tolerance to *Aspergillus* antigens in highly exposed subjects. IL-21 is an opponent of Th2 differentiation, inhibits IL-4-induced IgE formation [49,50,51], and can suppress the immune system through induction of IL-10 secretion in Th1-skewed stimulation environments [52,53,54]. Consequently, the strong induction of these cytokines in our organic farmer cohort points to a role of innate immune cells and their cytokines in the modulation of T-cellular immune networks in response to mold exposure.

Of note, flow cytometric detection of CD154^+^ AfuLy-reactive Th cells combined with the quantification of IFN-γ, IL-6, and MIP-1 α release had a classification accuracy of 92% between samples from occupationally exposed subjects and controls. This noteworthily high accuracy encourages further exploration of antigen-reactive immune end points as a bio-effect monitoring approach in subjects with occupational mold exposure [9,10]. However, despite the availability of improved WB-based protocols [18], flow cytometry is not feasible in most routine occupational healthcare settings and is difficult to standardize. Therefore, it would be desirable to entirely replace flow cytometry with WB-based cytokine release assays for bio-effect monitoring of (occupational) mold exposure. Optimized pools of well-defined *A. fumigatus* antigens might allow for improved accuracy of cytokine release assays without concomitant flow cytometry, but the optimal antigens or antigen combinations to efficiently track mold exposure remain elusive. Compared to the mycelial lysate, Aspf22, CatB, and CipC displayed markedly greater discriminatory power of Th1 (IFN-γ) and Th2 (IL-5) cytokine responses between the two cohorts in our ELISPOT screen. These results underline the potential benefits of defined protein antigens to improve cytokine monitoring approaches for occupational mold exposure and *Aspergillus*-associated hypersensitivity syndromes, encouraging further testing of these antigens in future studies.

Limitations to this study include the relatively small cohort sizes and lack of prospective validation of the most promising antigens and cytokine combinations in an independent cohort of occupationally exposed subjects. Furthermore, the inclusion of patients with hypersensitivity pneumonitis or mold-induced asthma resulting from occupational exposure would have greatly enhanced this study; however, such samples were not available to the investigators. In addition, as discussed above, Treg cells have important regulatory functions and play a major role in the tolerance of chronic mold exposure and prevention of *Aspergillus*-associated hypersensitivity diseases. The influence of high occupational exposure on Treg responses should therefore be evaluated more thoroughly in future studies. Lastly, we did not assess cytokine responses to other molds that are encountered in agricultural production, e.g., *Alternaria* species [55].

## 5. Conclusions

Our study provides new insights into the immune alterations induced by intensive or chronic occupational mold exposure. Specifically, we found co-expansion of Th1 and Th2 cell responses in chronically exposed subjects, enhanced expression of innate immune cell cytokines (IL-6, MIP-1α, and GM-CSF) that correlate with Th1 and Th2 responses, and discrimination of occupationally exposed subjects and controls by cytokine responses to *A. fumigatus* antigens Aspf22, CatB, and CipC. Altogether, these results encourage further prospective evaluation of mold-reactive cytokines responses as a potential bio-effect monitoring in occupational health and potential translation to other settings of extensive mold exposures, e.g., after flooding events.

## Data Availability

The data presented in this manuscript are available from the corresponding author upon reasonable request.

## Supplementary Materials

The following are available online at https://www.mdpi.com/article/10.3390/jof7090698/s1. Figure S1: Gating strategy and representative data set for flow cytometric analyses. Figure S2: Subjects with risk factors for mold exposure in their occupational or residential environment display significantly increased *A. fumigatus*-specific T-helper cell frequencies. Figure S3: Heatmaps of median and individual cytokine responses (WB-based Luminex assay) to *A. fumigatus* antigens in samples from organic farmers and controls. Figure S4: Volcano analysis of cytokine responses to *A. fumigatus* antigens identifies a selection of cytokine markers with significant or modest discriminatory power between organic farmers and controls. Figure S5: T-cellular and innate immune cell-derived cytokine responses to AfuLy are strongly correlated in our cohort of organic farmers and controls. Figure S6: Cytokine concentrations detectable by WB-based Luminex analysis modestly differentiate immune responses of organic farmers and controls. Table S1: Oligonucleotides used for the generation of recombinant *A. fumigatus* proteins. Table S2: Preparation of stimulation tubes for whole-blood stimulation.
