# Supplementary material for: Chronic Occupational Mold Exposure Drives Expansion of Aspergillus-Reactive Type 1 and Type 2 T-Helper Cell Responses
Source: J Fungi (Basel). 2021 Aug 27;7(9):698. doi: 10.3390/jof7090698 (PMC8471116; doi:10.3390/jof7090698)
Supplement: Supplementary file 1 [file jof-07-00698-s001.zip › jof-1321640-supplementary.pdf]

Journal of *Fungi*

MDPI

Chronic occupational mold exposure drives expansion of *Aspergillus*-reactive type 1 and type 2 T-helper cell responses

Supplementary Figures and Tables

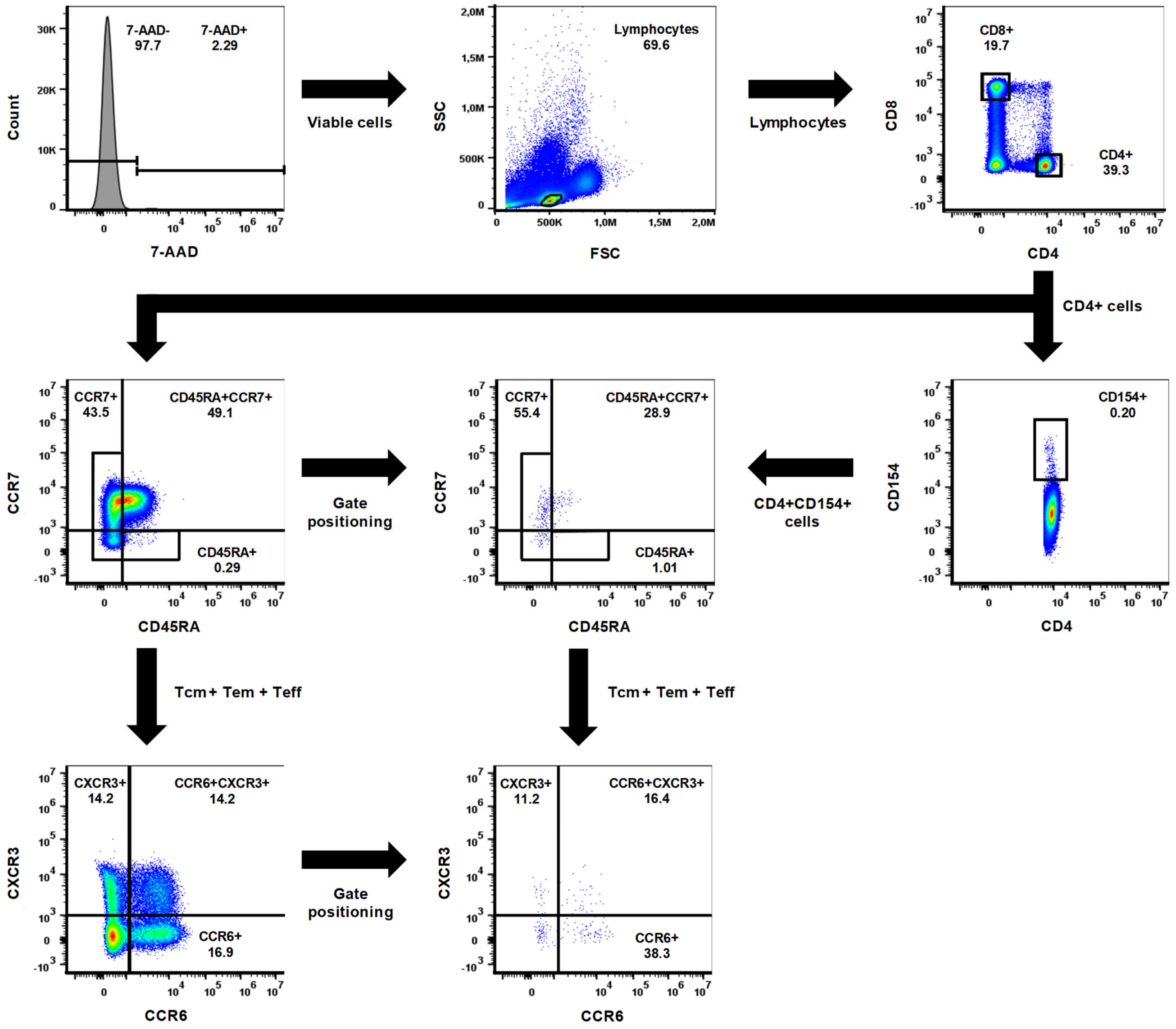

Figure S1. Gating strategy and representative data set for flow cytometric analyses. 7-Aminoactinomycin D (7-AAD)-positive (dead) cells were excluded, lymphocytes were identified by light scatter properties, and $CD4^+$ T-helper (Th) cells were gated. Effector Th cells (Teff; $CD4^+CD45RA^+CCR7^-$), effector memory Th cells (Tem; $CD4^+CD45RA^-CCR7^-$), central memory Th cells (Tcm; $CD4^+CD45RA^-CCR7^+$), and naive Th cells ($CD4^+CD45RA^+CCR7^+$) were differentiated among $CD4^+$ cells. An additional gate including the Tcm, Tem, and Teff subsets was positioned in order to exclude naive Th cells from subsequent Th1/Th2/Th17 cell differentiation. Thereafter, the non-naïve $CD4^+$ cells were subdivided into Th1 cells ($CD4^+CCR6^-CXCR3^+$), Th2 cells ($CD4^+CCR6^-CXCR3^-$), Th17 cells ($CD4^+CCR6^+CXCR3^-$), and others including Th1/Th17-type cells ($CD4^+CCR6^+CXCR3^+$). Antigen-reactive cells among the total $CD4^+$ Th population were identified by CD154 expression. The CD45RA/CCR7 and CCR6/CXCR3 gates were then transferred to the $CD4^+CD154^+$ population in order to define

Journal of Fungi

memory/effector phenotypes and polarization of the antigen-reactive Th cells. Abbreviations: CD = cluster of differentiation, CCR = C-C motive chemokine receptor, CXCR = C-X-C motive chemokine receptor.

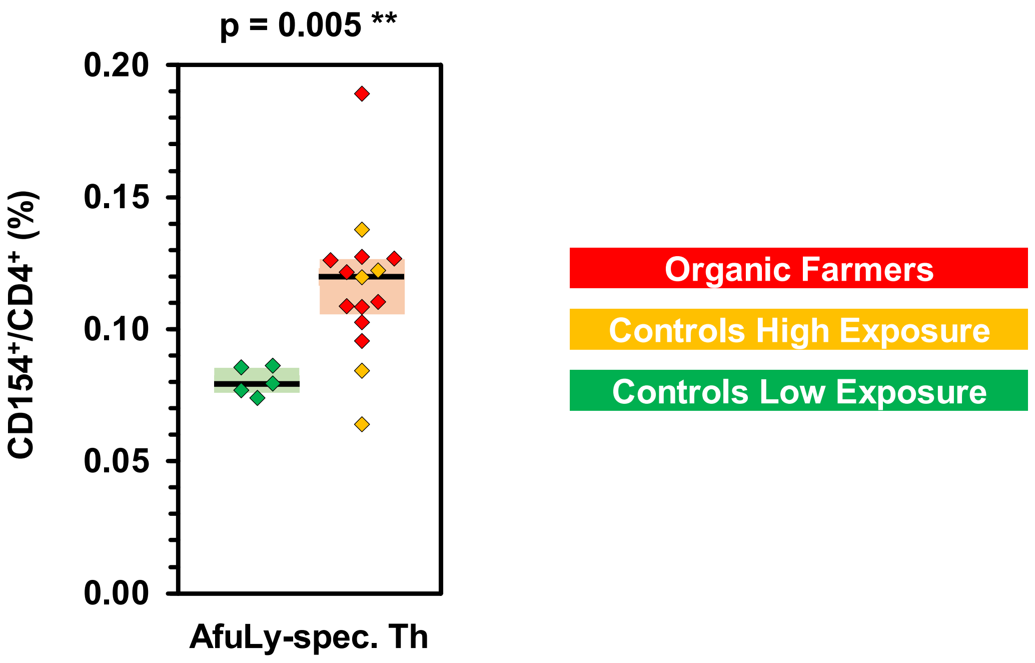

Figure S2. Subjects with risk factors for mold exposure in their occupational or residential environment display significantly increased *A. fumigatus*-specific T-helper cell frequencies. PBMCs from 10 organic farmers (red), 5 control subjects with high residential mold exposure (yellow), and 5 control subjects reporting no significant risk factors for occupational or residential exposure (green) were stimulated with *A. fumigatus* mycelial lysate (AfuLy). Frequencies of $CD154^+$ cells among ($CD4^+$) T-helper cells were determined by flow cytometry. Unlike in Fig. 1a, subjects are grouped according to our previously reported classification of mold exposure profiles (Wurster et al., 2017, Mycoses; Page et al., 2018, Intern J Med Microbiol). Individual background-adjusted results, medians (black bars), and inter-quartile ranges (colored boxes) are shown. Two-sided Mann-Whitney-U-test.

Journal of Fungi

MDPI

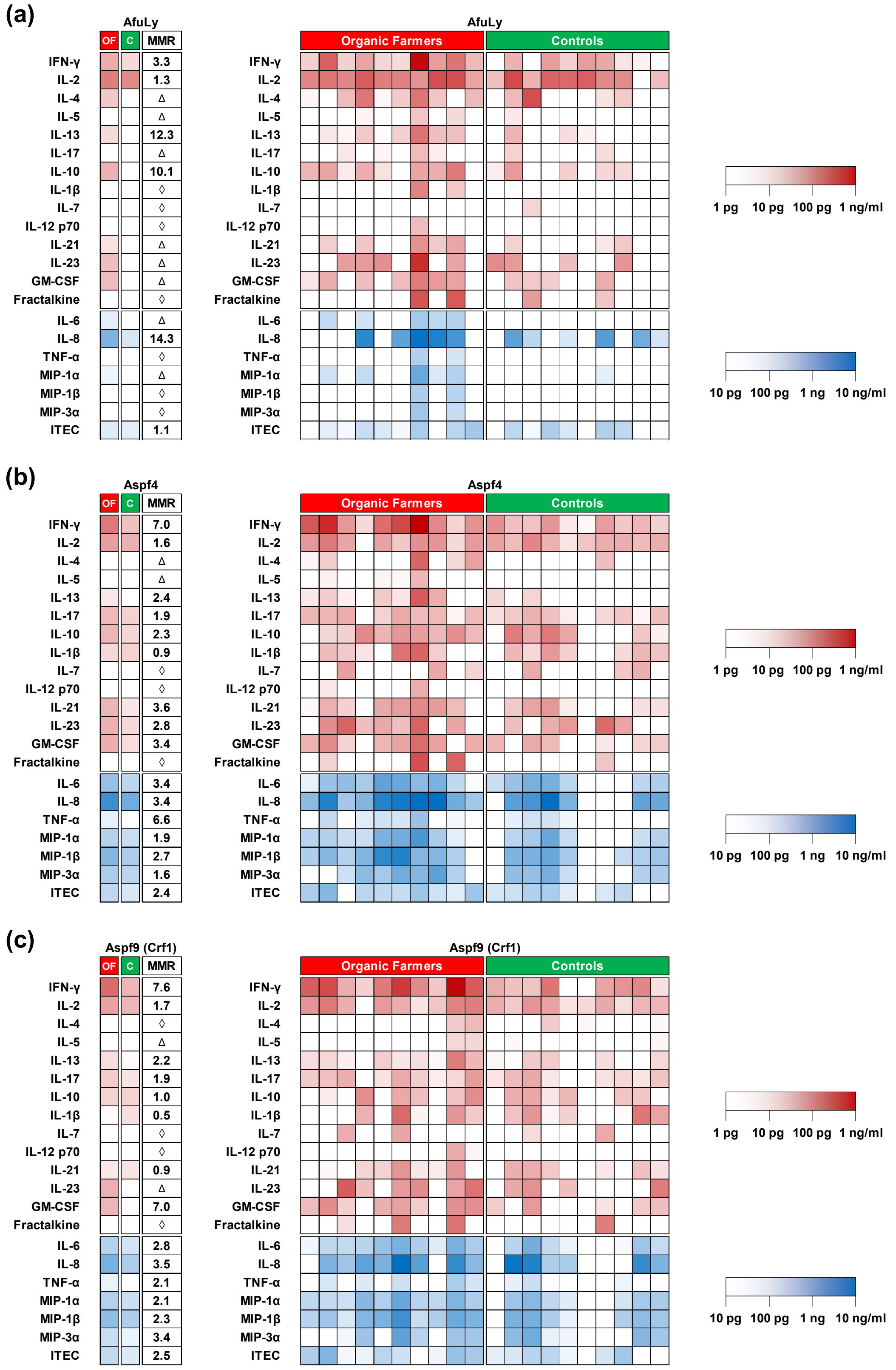

Figure S3. Heatmaps of median and individual cytokine responses (whole blood-based Luminex assay) to *A. fumigatus* antigens in samples from organic farmers and controls. The heatmaps summarize median and individual background-

Journal of Fungi

adjusted cytokine concentrations in organic farmers (“OF”) and control subjects (“C”) depending on the *A. fumigatus* antigen used for stimulation. The numeric values in the MMR column represent median-to-median ratios between the two cohorts, with values >1.0 indicating greater median cytokine concentrations in the organic farmer cohort. Δ denotes infinite median-to-median ratios (median = 0 pg/mL in the control cohort). ◊ denotes undefined median-to-median ratios (median = 0 pg/mL in both cohorts). AfuLy = *A. fumigatus* mycelial lysate.

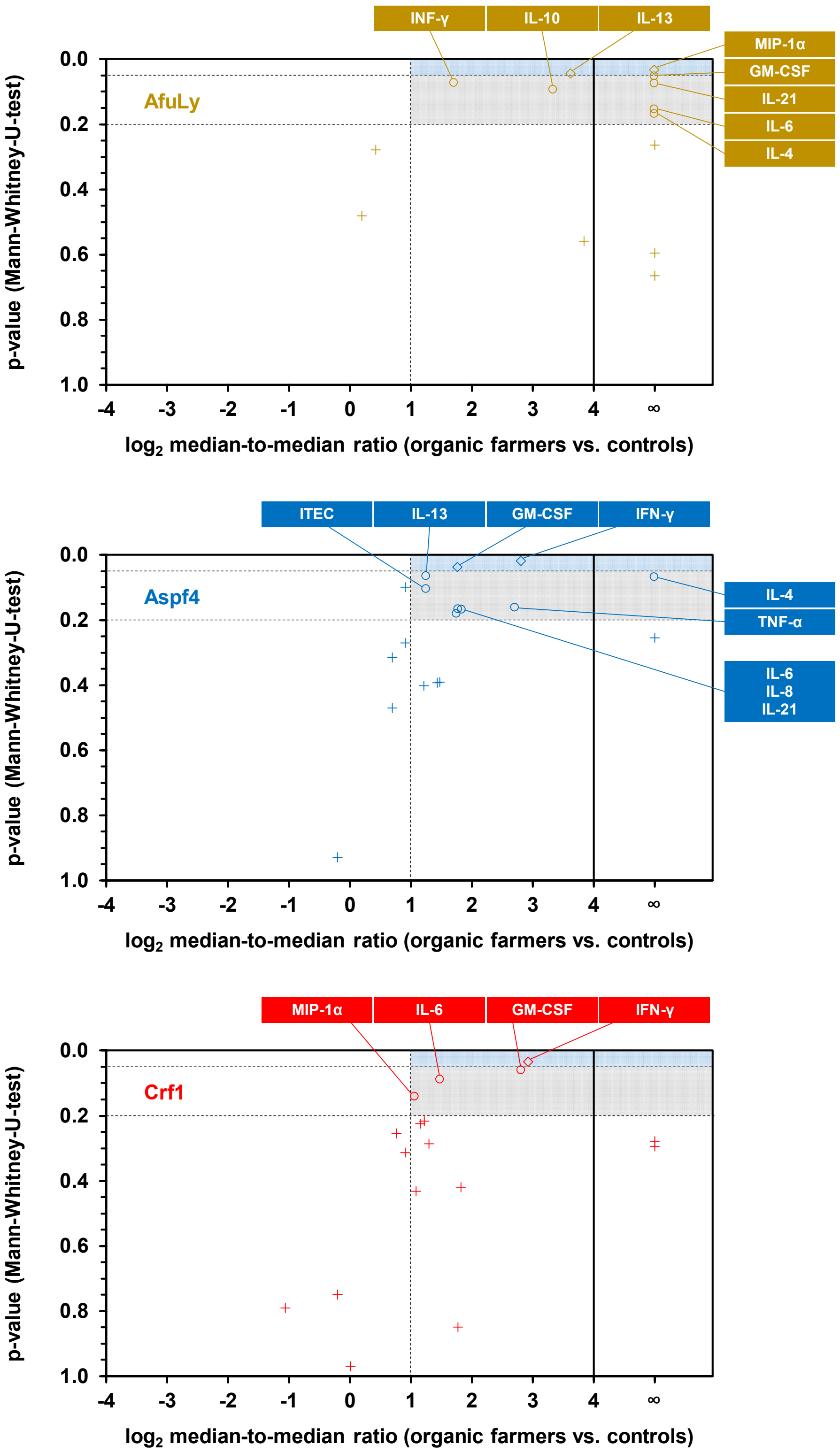

Figure S4. Volcano analysis of cytokine responses to *A. fumigatus* antigens identifies a selection of cytokine markers with significant or modest discriminatory power between organic farmers and controls. The volcano plots compare log_2-transformed ratios of median concentrations for each antigen/cytokine combination in organic farmers and controls (x-values)

Journal of Fungi

with the corresponding p-values (Mann-Whitney-U-test, y-values). Antigen/cytokine pairs are classified as insignificant ("+" symbols, median-to-median ratio < 2 and/or p > 0.2), potentially significant (circles, median-to-median ratio > 2 and 0.05 < p < 0.2), or significant (diamonds, median-to-median ratio > 2 and p < 0.05). Cytokine responses with medians of 0 pg/mL in both cohorts are not displayed.

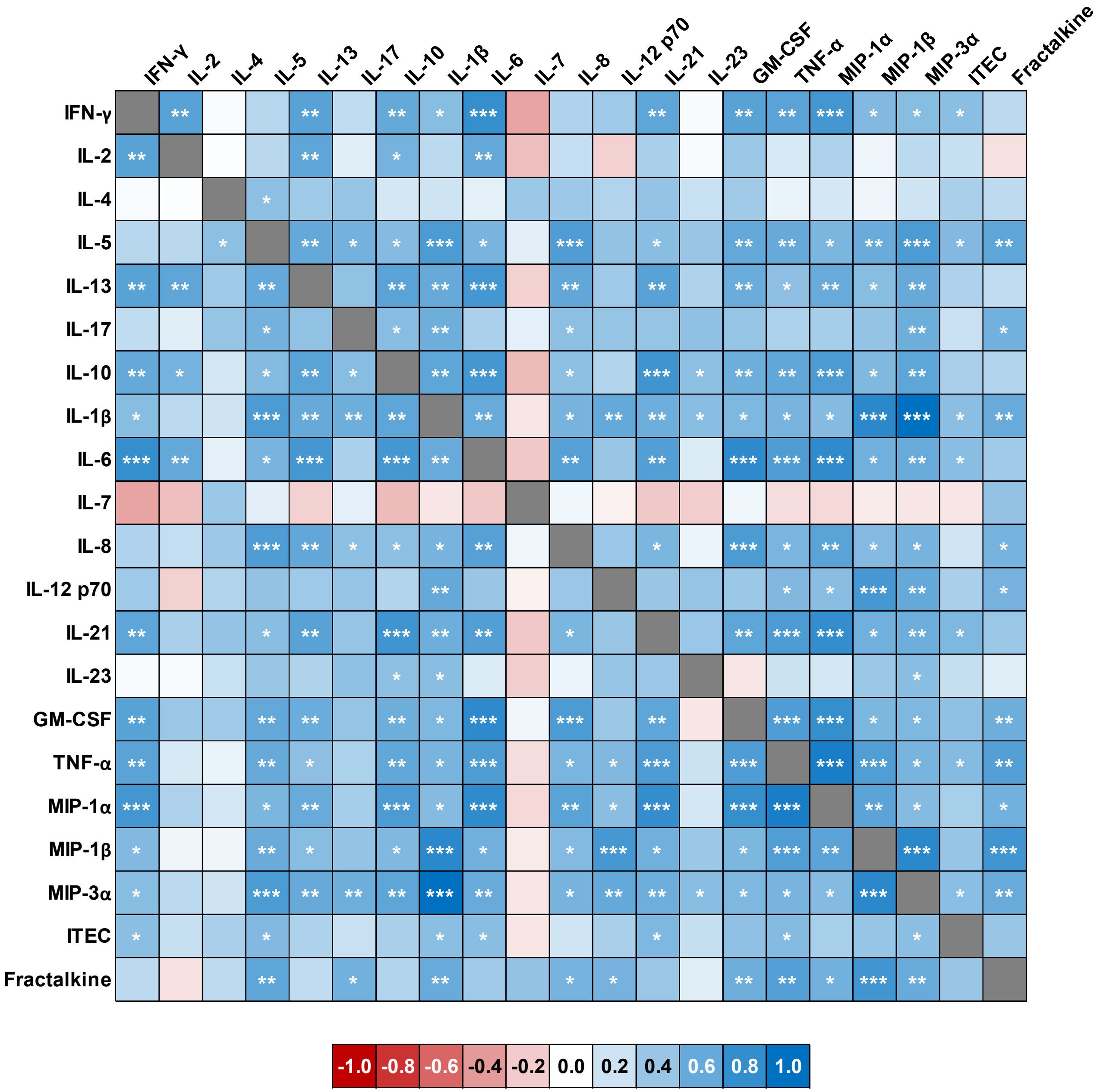

Figure S5. T-cellular and innate immune cell-derived cytokine responses to *A. fumigatus* antigens are strongly correlated in our cohort of organic farmers and controls. Heatmap of Spearman's rank correlation coefficients of individual cytokine concentrations elicited by *A. fumigatus* mycelial lysate (whole blood-based Luminex assay). FDR-corrected significance of Spearman coefficients is indicated by asterisks. * p < 0.05, ** p < 0.01, *** p < 0.001.

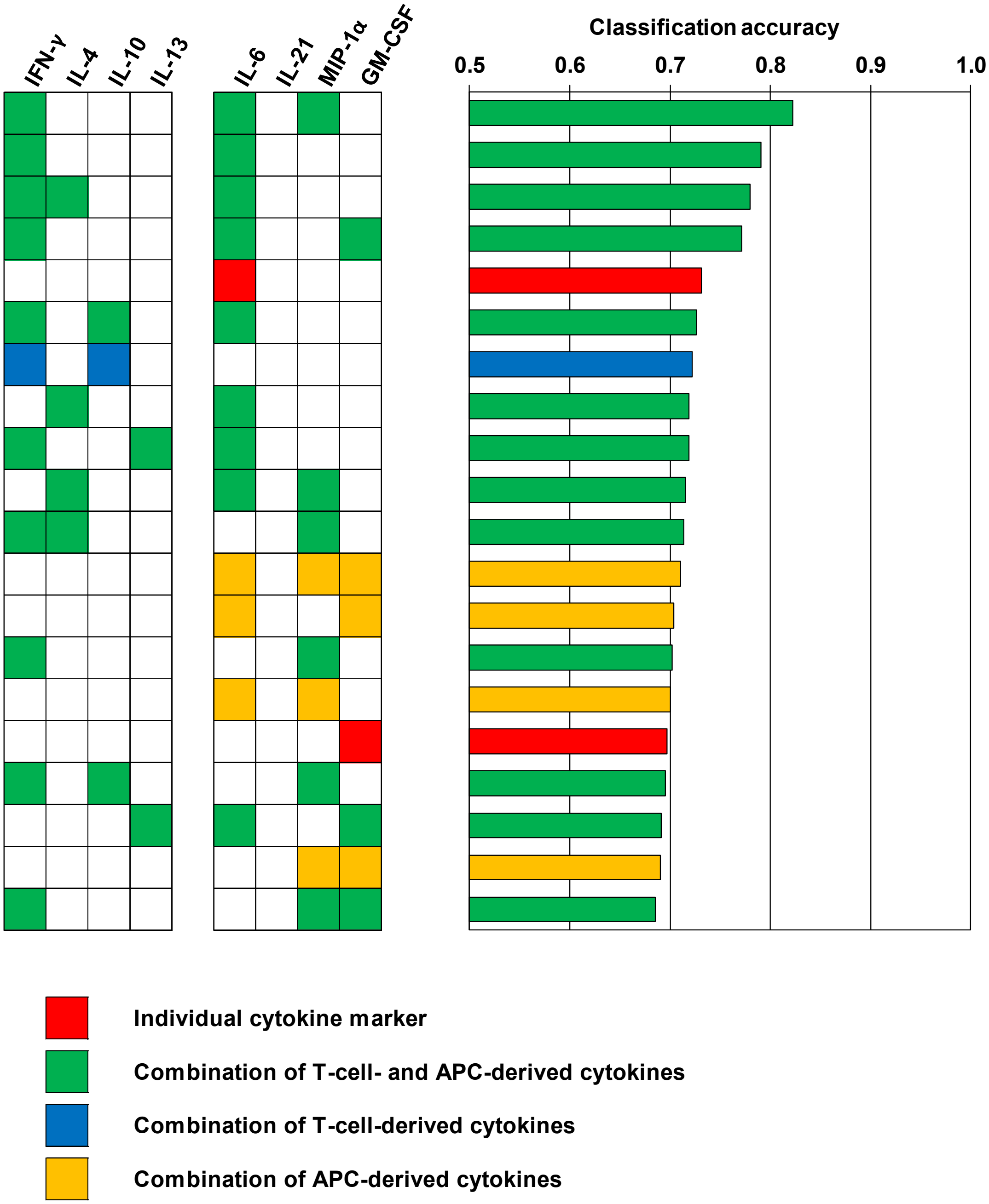

Figure S6. Cytokine concentrations detectable by whole blood-based Luminex analysis modestly differentiate immune responses of organic farmers and controls. The classification accuracy of combinations of up to 3 AfuLy-induced cytokines responses was determined by a random forest algorithm. The top 20 combinations with the highest classification accuracy between organic farmers and controls are shown. Only cytokine markers passing the pre-filtering step (Fig. S4) based on their relative induction in the two cohorts (median-to-median ratio > 2.0) and p-values ($p < 0.2$) were considered. Abbreviations: AfuLy = *A. fumigatus* mycelial lysate, APC = antigen presenting cells.

Journal of *Fungi*

MDPI

Table S1. Oligonucleotides used for the generation of recombinant *A. fumigatus* proteins.

Designation	Systematic name	Localization	Forward primer Restriction sites are indicated by bold fonts.	Reverse primer Restriction sites are indicated by bold fonts.
Aspf1 (mitogillin)	Afu5g02330	secreted	Aspf1-BamHI-FOR GCA**GGATCC**ACCTGGACATGCATCAAC	Aspf1-HindIII-REV GCA**AAGCTT**CTAATGAGAACACAGTCT
Aspf3	Afu6g02280	cytosolic	Aspf3-BamHI-FOR CT**GGATCC**ATGTCTGGACTCAAGGCCGGT	Aspf3-HindIII-REV GCA**AAGCTT**TTACAGGTGCTTGAGGACGGT
Aspf4	Afu2g03830	secreted	Aspf4-BamHI-FOR CA**GGATCC**CACGAGCGCCGCCACCTCCAC	Aspf4-HindIII-REV GCA**AAGCTT**CTACTCCTTGTAGTCGAGGTT
Aspf6 (MnSOD)	Afu1g14550	mitochondrial	Aspf6-BamHI-FOR AG**GGTACC**ATGTCACAGCAATACACG	Aspf6-HindIII-REV GCA**AAGCTT**CTACAGCTTCATGAATGG
Aspf8	Afu2g10100	cytosolic	Aspf8-BamHI-FOR GC**GGATCC**TCCTCTGAGGATGTCAAGGCC	Aspf8-HindIII-REV AGC**AAGCTT**TTAGTCGAAAAGACCGAAGCC
Aspf9	Afu1g16190	secreted / cell wall	Aspf9-BamHI-FOR CT**GGATCC**AAGACCTGCCCGCCCAACAAG	Aspf9-HindIII-REV ACT**AAGCTT**TTAGAATGCCAACACGGCAG
Aspf22 (enolase)	Afu6g06770	cytoplasm	Eno-BamHI-FOR CG**GGATCC**ATGCCTATCTCCAAGATC	Eno-PstI-REV GCG**CTGCAG**TTACAGGTTGACGGCAGT
CatB (catalase)	Afu3g02270	secreted / cell wall	Cat1-BamHI-FOR CA**GGATCC**GTATGTCCCTATATGACC	Cat1-KpnI-REV AG**GGTACC**CTCGATTGCATCGTGCAA
CipC	Afu5g09330	cytoplasm	CipC-BamHI-FOR CA**GGATCC**ATGGCTTGGGGCTGG	CipC-HindIII-REV GCA**AAGCTT**TTACCAACGGTCGAC
CsnB (chitosanase B)	Afu4g01290	secreted	CsnB-BamHI-FOR TA**GGATCC**TATAATTTGCCCAACAAC	CsnB-HindIII-REV GTA**AAGCTT**CTATGCTTTCAAACCAGC
Hly (hemolysin)	Afu3g00590	secreted	Hly-BamHI-FOR CA**GGATCC**ATGGCATCGGTCCAAGCT	Hly-KpnI-REV AG**GGTACC**TCAGCGCTTCCTTCCAAC

Pst2	Afu1g02820	cytoplasm	Pst2-BamHI-FOR CA**GGATCC**TACTCCATGTACGGCCAC	Pst2-HindIII-REV GCA**AAGCTT**TCACGCGAAGTTGACCTT

Abbreviations: Aspf = *Aspergillus fumigatus* allergen, BamHI = *Bacillus amyloliquefaciens* restriction endonuclease HI, CipC = concanamycin-induced protein C, Eno = enolase, FOR = forward, HindIII = *Haemophilus influenzae* restriction endonuclease DIII, KpnI = *Klebsiella pneumoniae* restriction endonuclease I, MnSOD = manganese superoxide dismutase, PstI = *Providencia stuartii* restriction endonuclease I, Pst2 = oxidative stress response protein 2, REV = reverse.

Table S2. Preparation of stimulation tubes for whole blood stimulation. .

	**α-CD28**	**α-CD49d**	**AfuLy**	**Protein antigens**	**PHA**
**Concentration in the ready-to-use stimulation tube**	2 µg/mL	2 µg/mL	100 µg/mL	60 µg/mL	20 µg/mL
**Final concentration after injection of 500 µL whole blood**	1 µg/mL	1 µg/mL	50 µg/mL	30 µg/mL	10 µg/mL
**Unstimulated background control**	X	X			
**AfuLy stimulation**	X	X	X		
***Aspergillus* protein stimulation**	X	X		X	
**Positive control**					X

X indicates that the reagent was used for the respective stimulation mix. The volume of the stimulation mix in the ready-to-use tubes was adjusted to 500 µL with RPMI 1640. Abbreviations: α = anti, AfuLy = *Aspergillus fumigatus* mycelial lysate, CD = cluster of differentiation, PHA = phytohemagglutinin, RPMI = Roswell Park Memorial Institute.
